# Pilot test of an interactive obesity treatment approach among employed adults in a university medical billing office

**DOI:** 10.1186/s40814-020-00599-w

**Published:** 2020-04-28

**Authors:** Rachel G. Tabak, Jaime R. Strickland, Bridget Kirk, Ryan Colvin, Richard I. Stein, Hank Dart, Graham A. Colditz, Ann Marie Dale, Bradley A. Evanoff

**Affiliations:** 1 The Brown School, Washington University in St. Louis, 1 Brookings Dr, St. Louis, MO 63130 USA; 2 Department of Medicine, Division of General Medical Sciences, Washington University School of Medicine, 4523 Clayton Avenue, Campus Box 8005, St. Louis, MO 63110 USA; 3 Center for Human Nutrition, Department of Medicine, Washington University School of Medicine, 4523 Clayton Avenue, Campus Box 8031, St. Louis, MO 63110 USA; 4 Division of Public Health Sciences, Washington University School of Medicine, 660 South Euclid Avenue, Campus Box 8100, St Louis, MO 63110 USA; 5 Division of Public Health Sciences and Alvin J. Siteman Cancer Center, Department of Surgery, Washington University School of Medicine, Washington University in St. Louis, 660 South Euclid Avenue, Campus Box 8100, St. Louis, MO 63110 USA

## Abstract

**Background:**

There is a need for workplace programs promoting healthy eating and activity that reach low-wage employees and are scalable beyond the study site. Interventions designed with dissemination in mind aim to utilize minimal resources and to fit within existing systems. Technology-based interventions have the potential to promote healthy behaviors and to be sustainable as well as scalable. We developed an interactive obesity treatment approach (iOTA), to be delivered by SMS text messaging, and therefore accessible to a broad population. The aim of this pilot study was to evaluate participant engagement with, and acceptability of, this iOTA to promote healthy eating and activity behaviors among low-wage workers with obesity.

**Methods:**

Twenty participants (self-reporting body mass index ≥ 30 kg/m^2^) of a single workgroup employed by a university medical practice billing office had access to the full intervention and study measures and provided feedback on the experience. Height and weight were measured by trained research staff at baseline. Each participant was offered a quarterly session with a health coach. Measured weight and a self-administered survey, including dietary and activity behaviors, were also collected at baseline, 3, 6, 12, 18, and 24 months. Participant engagement was assessed through responsiveness to iOTA SMS text messages throughout the 24-month pilot. A survey measure was used to assess satisfaction with iOTA at 3 months. Due to the small sample size and pilot nature of the current study, we conducted descriptive analyses. Engagement, weight change, and duration remaining in coaching are presented individually for each study participant.

**Results:**

The pilot was originally intended to last 3 months, but nearly all participants requested to continue; we thus continued for 24 months. Most (14/20) participants remained in coaching for 24 months. At the 3-month follow-up, eight (47%) of the remaining 17 participants had lost weight; by 24 months, five (36%) of the remaining 14 participants had lost weight (one had bariatric surgery). Participants reported very high satisfaction.

**Conclusions:**

This pilot provides important preliminary results on acceptability and participant engagement with iOTA, which has significant potential for dissemination and sustainability.

## Background

Healthy eating and activity are important to prevent obesity and chronic diseases such as diabetes. Low-wage workers bear a disproportionate share of the chronic disease burden [1–6] yet have more limited access to workplace resources that promote healthy eating and activity [7, 8]. It is therefore critical that programs to support health-promoting behaviors be easy to access and acceptable to this population. Often, even when such resources are available in a worksite, low-wage workers are less likely to engage with them due to a number of barriers including acceptability and social support [8–19].

Despite the benefits of existing workplace programs that promote healthy eating and activity, limitations remain [20–25], particularly in reaching low-wage employees. Further, there has been limited ability to scale effective programs beyond the study site [26]. Interventions designed with dissemination in mind aim to utilize minimal resources and to fit well within existing systems [27].

Technology-based interventions have the potential to promote healthy behaviors and to be sustainable as well as scalable [23, 28, 29] A number of technological methods have been explored to support health behaviors related to obesity [23, 29–31]. Many of these methods, however, require specific hardware/software and/or time on the part of participants, making them out of reach for or unappealing to individuals with limited resources. SMS (short message service) text messaging is ubiquitous, inexpensive, standardized across mobile phone platforms, and does not require additional software, applications, mobile phone data plan, or internet access, making it an ideal system to reach low-wage workers [32–37]. In response to this, we developed an interactive obesity treatment approach (iOTA), designed to be delivered by SMS text messaging, and therefore accessible to a broad population. Development of this SMS text-based iOTA has been described previously [38]. Briefly, this intervention was adapted from an evidence-based weight loss program [39, 40] and includes a baseline meeting with a health coach at which three behavior change goals are selected. Each participant then receives interactive, automatic text messages which include weekly weight and goal achievement check-ins as well as one behavioral tip per week for each goal and periodic motivational messages. iOTA goals are fully described elsewhere [41]; sample behavioral tip messages include: “Don’t tempt yourself - Empty the fridge of sugary drinks. And keep low-calorie choices handy, like water and unsweetened coffee or tea.” and “Don’t worry. There are healthy fast food options. Try grilled chicken, turkey sandwiches without cheese & mayo, and veggie stir-fry with brown rice.” Additional coaching sessions to reflect on goal progress and brainstorm ways to address barriers to goal achievement occur quarterly. At these sessions, coaches provide education and resources related to chosen goal topics, and participants have the option to change goals. Pilot testing of this iOTA was conducted to understand how participants engage with the intervention, participant satisfaction, and to summarize lessons learned for larger-scale evaluation.

This study fills an important gap in reporting on engagement with an SMS text-based workplace intervention designed for dissemination and sustainability. The aim of this pilot study was to evaluate participant engagement with and acceptability of this iOTA to promote healthy eating and activity behaviors among low-wage workers with obesity.

## Methods

### Study design

This pilot study allowed for preliminary evaluation of the iOTA and measures used in assessment of behavior change among low-wage employees. Participants in this pilot had access to the full intervention (weekly SMS text messaging and quarterly health coach sessions, where they received handouts related to their discussions) and study measures and provided feedback on the experience. Participants provided informed consent to participate in the initial pilot and additional consent to extend the pilot. To compensate for their time completing data collection, participants received $25 at baseline, $50 at 3 months, $20 at 6 months, $30 at 12 months, $20 at 18 months, and $30 at 24 months. The study was approved by the Human Research Protection Office at Washington University in St. Louis (#201609029).

### Setting and participants

A workgroup from a university medical practice billing office was recruited to participate in the pilot study. Given the focus of the intervention on low-wage employees, this workgroup was selected to mirror the inclusion criteria employed in the larger trial to evaluate iOTA, including a high proportion of lower wage workers. Recruitment criteria for the larger trial are described fully elsewhere [41]. From this group, employees self-reporting a body mass index (BMI) greater than 30 kg/m^2^ were invited to participate and were screened for exclusion criteria: currently pregnant or nursing, bariatric surgery in the last 12 months, current cancer diagnosis, or planning to leave the work unit within the next month. Based on previous experience with pilot testing interventions, 20 participants was thought to be a reasonable recruitment goal that would be logistically feasible and still yield enough data to understand participant engagement with and acceptability of iOTA, as well as to determine major changes needed to the intervention. Since the goal for the pilot was 20 participants, the first 20 eligible participants to volunteer were included, though more than 20 expressed interest. Height and weight were measured by trained research staff at baseline to verify BMI prior to enrollment.

### Measures

#### Program engagement

Administration of the iOTA via an interactive, automated SMS text-messaging program allowed for assessment of participant engagement with the program, based on responsiveness to iOTA text messages. The percent of weeks during which participants responded to text message prompts (to report progress on weight and goal attainment) was assessed throughout each participant’s enrollment in the pilot. As part of the iOTA protocol, if a participant reported meeting their goal 3 weeks in a row, they were offered the opportunity (through the automated SMS texting system) to make the goal more challenging or maintain their original goal. The success rate for each participant was explored based on their self-report of goal achievement across all weeks. These data were collected throughout the 24-month pilot.

#### Eating and activity behavior

Dietary behaviors were assessed using the Rapid Eating Assessment for Participants (REAP-S) measure [42, 43]. Physical activity was measured with the job-related and leisure time domains of the International Physical Activity Questionnaire (IPAQ) [44, 45]. These self-report measures were collected in baseline and follow-up surveys at 3, 6, 12, 18, and 24 months. The baseline survey also included demographics and psychosocial supports for eating and physical activity.

#### Height and weight

Height and weight were measured by trained research staff at baseline. These staff also measured each participant’s weight at each quarterly coaching session; 3, 6, 12, 18, and 24-month weights are presented, as they correspond to survey data collection.

#### Satisfaction

A survey measure designed specifically for the study was used to assess satisfaction with and suggested modifications for iOTA at 3 months. Questions with Likert-type responses (1 = strongly disagree, 2 = disagree, 3 = neutral, 4 = agree; 5 = strongly agree) asked about perceptions of the overall program, the health coaching interaction, the text messaging, and overall perceived benefits to participation. Participants also had the opportunity to respond to open-ended questions asking about their favorite parts of iOTA, what they would change, and to provide any additional feedback.

### Analysis

Due to the small sample size and pilot nature of the current study, we conducted descriptive analyses. Engagement, weight change, and length of time remaining in coaching are presented individually for each study participant. The percent of participants reporting any, 3%, or 5% weight loss, along with mean weight change in pounds, are presented at each time point. Eating behaviors were summarized with the mean REAP-S score, and the number and percent of participants are reported for several individual items (e.g., often skip breakfast), which were dichotomized to “often” (usually, often) and “not often” (sometimes, rarely, never). Finally, several items ask participants to report the number of days in a typical week a behavior was completed (e.g., bring food from home); these were summarized to mean number of days per week. Activity is summarized both continuously (mean of the total minutes per week walking, doing moderate activity, and/or doing vigorous activity) and in terms of the number and percent of participants reporting they met the Centers for Disease Control and Prevention physical activity recommendation of 150 min moderate to vigorous physical activity (MVPA) per week. To better understand how pilot participants were using the iOTA, the goals selected and percent of weeks participants reported successfully achieving each goal is summarized as are means for items assessing program satisfaction.

## Results

The pilot was originally intended to last 3 months, but nearly all participants requested that the pilot study continue after that time; we thus continued for 21 more months (24 months total, the duration of the full clinical trial testing the iOTA intervention, which incorporated the feedback from the current pilot) [41]. Participants were predominantly female (85%). Few participants had college degrees (15%). Among those who reported income, half made less than $21 per hour; more than half of participants reported a health condition potentially related to obesity (Table 1).

**Table 1:** Distribution of baseline characteristics, iOTA pilot participants

	Overall
	*n* = 20
Age category in years	
30–40	7 (35.0)
41–50	4 (20.0)
51–60	9 (45.0)
Gender = female	17 (85.0)
Race = White/Caucasian	10 (50.0)
Education	
High school/GED	2 (10.0)
Some college	15 (75.0)
College-Bachelor’s degree	3 (15.0)
Hourly pay	
$10.0–15.9	1 (5.0)
$16.0–20.9	7 (35.0)
$21.0–25.9	4 (20.0)
$26.0–30.9	0 (0.0)
$31.0+	4 (20.0)
Not reported	4 (20.0)
Marital status	
Never married	3 (15.0)
Married	13 (65.0)
Divorced/separated	2 (10.0)
Member of an unmarried couple	2 (10.0)
BMI, kg/m^2^	35.6 (33.7, 40)
BMI by category	
Normal weight (BMI < 25.0)	0 (0.0)
Overweight (BMI 25.0–29.9)^a^	1 (5.0)
Obese (BMI ≥ 30)	19 (95.0)
SF-8 physical component score	48 (41, 53.4)
SF-8 mental component score	47.1 (38.3, 56.2)
Diabetes dx in past 6 months	1 (5.0)
Hypertension dx in past 6 months	10 (50.0)
Hypercholesterolemia dx in past 6 months	6 (30.0)
Have ≥ 1 conditions listed above	11 (55.0)
Have ≥ 2 conditions listed above	6 (30.0)
Usual hours worked per week	40 (40, 40)
Work non-day shifts	1 (5.0)
Coworker support scale^b^	8.5 (7.8, 10.2)
Supervisor support scale^b^	10.5 (8, 12)
Participated in ≥ 1 wellness program in the workplace	17 (85.0)

Presented are *n* (%) for categorical variables, median (25th, 75th percentile) for continuous variables

^a^One participant lost weight between the self-report assessment of eligibility and the baseline weight and thus moved to overweight status (BMI = 29.2, initial, BMI based on self-reported weight = 30.4)

^b^Range 0–12, with higher scores indicating more support

Overall, there were very high rates of engagement across the 24 months (Table 2). Two participants discontinued participation prior to the first follow-up session. Two participants completed only the originally described 3 months while most participants elected to remain in coaching for extended time: one to 9 months, another one continuing to 12 months, and the remaining 14 participants continued to participate in coaching for the entire 24 months. Of those that discontinued, two participants left the workgroup (one prior to 3 months and one after 9 months), so we were not able to continue the follow-up after they left and were dropped from study participation. From Table 2, it appears that participants continued to respond to prompts for most weeks leading up to the time they stopped participating in coaching or the end of the 24-month pilot. Table 2 also depicts the range of weight change among participants (excluding a 97-pound weight loss by the participant who had bariatric surgery during the study), from a 41-pound weight loss to a 44-pound weight gain.

**Table 2:** Participant responsiveness over time and individual weight change (lbs)

	Weekly text message response, (*N* responses, % of possible weeks to respond))					Last coaching session attended	Baseline weight	Final weight	Final weight change
Participant	3 months	6 months	12 months	18 months	24 months				
1	11 (84.6)	24 (92.3)	50 (96.1)	72 (92.3)	85 (81.7)	24 months	182.8	153.0	− 29.8
2^a^	12 (92.3)	25 (96.1)	50 (96.1)	75 (96.1)	95 (91.3)	24 months	291.6	194.2	− 97.4
3	12 (92.3)	25 (96.1)	50 (96.1)	70 (89.7)	92 (88.4)	24 months	221.0	229.4	+ 8.4
4	12 (92.3)	25 (96.1)	50 (96.1.0)	75 (96.1)	100 (96.1)	24 months	235.8	252.1	+ 16.3
5	13 (100.0)	26 (100.0)	52 (100.0)	78 (100.0)	104 (100.0)	24 months	233.4	242.0	+ 8.6
6	12 (92.3)	12 (46.1)	12 (23.0)	12 (15.3)	12 (11.5)	3 months	198.0	188.0	− 10.0
7	11 (84.6)	12 (46.1)	12 (23.0)	12 (15.3)	12 (11.5)	3 months	281.8	298.2	+ 16.4
8	12 (92.3)	24 (92.3)	45 (86.5)	45 (57.6)	45 (43.2)	9 months	235.0	248.0	+ 13.0
9	13 (100.0)	26 (100.0)	49 (94.2)	72 (92.3)	96 (92.3)	24 months	250.4	294.4	+ 44.0
10	13 (100.0)	26 (100.0)	52 (100.0)	77 (98.7)	101 (97.1)	24 months	251.2	256.8	+ 5.6
11	13 (100.0)	26 (100.0)	52 (100.0)	75 (96.1)	99 (95.1)	24 months	250.2	272.8	+ 22.6
12^b^	5 (38.4)	5 (19.2)	5 (9.6)	5 (6.4)	5 (4.8)	n/a	237.0	n/a	n/a
13	11 (84.6)	19 (73.0)	27 (51.9)	41 (52.5)	45 (43.2)	24 months	188.0	207.8	+ 19.8
14	8 (61.5)	15 (57.6)	27 (51.9)	29 (37.1)	29 (27.8)	12 months	232.6	245.8	+ 13.2
15^b^	9 (69.2)	11 (42.3)	11 (21.1)	11 (14.1)	11 (10.5)	n/a	182.6	n/a	n/a
16	7 (53.8)	10 (38.4)	23 (44.2)	42 (53.8)	55 (52.8)	24 months	239.4	253.4	+ 14.0
17	12 (92.3)	25 (96.1)	51 (98.0)	77 (98.7)	103 (99.0)	24 months	203.2	215.8	+ 12.6
18	12 (92.3)	24 (92.3)	43 (82.6)	66 (84.6)	77 (74.0)	24 months	355.0	352.8	− 2.2
19	13 (100.0)	25 (96.1)	51 (98.0)	76 (97.4)	100 (96.1)	24 months	170.0	154.5	− 15.5
20	13 (100.0)	26 (100.0)	49 (94.2)	73 (93.5)	93 (89.4)	24 months	222.0	180.6	− 41.4

Proportion of weekly SMS text messages to which participant responded, grouped by 3-month intervals corresponding to coaching sessions. A response was defined as participant report of weekly weight or goal completion status

^a^Participant had gastric bypass surgery

^b^Participant did not attend any follow-up coaching sessions

Within the initial pilot phase, eight (47%) of the original 17 participants with 3-month data had lost weight (Table 3). By 24 months, four (31%) of the remaining 13 participants had lost weight, and three of these (23%) had lost 5% of their body weight. The median weight change was a gain at all time points (8.6, 25th, 75th percentile − 2.2, 16.3 pounds at 24 months), but among those who lost weight, the median weight loss increased from 3 to 24 months.

**Table 3:** Weight change (lbs) from baseline

Measurement time point	Lost any weight	Lost ≥ 3% of weight	Lost ≥ 5% of weight	Weight change^a^, lbs	Weight loss^b^, lbs	Maximum weight loss, lbs
3 months (*N* = 17)	8 (47.1)	3 (17.6)	1 (5.9)	+ 2.2 (− 5.6, 5)	− 6.3 (− 7.8, − 2.1)	− 14.2
6 months (*N* = 15)	6 (40.0)	2 (13.3)	1 (6.7)	+ 1.8 (− 1.6, 7)	− 3.7 (− 8, − 1)	−10.6
12 months (*N* = 14)	4 (28.6)	2 (14.3)	2 (14.3)	+ 7.4 (0.1, 12.6)	− 9.8 (− 19.1, − 4.3)	− 34.6
18 months (*N* = 13)	6 (46.2)	2 (15.4)	2 (15.4)	+ 4.8 (− 4.5, 12.3)	− 5.2 (− 18.9, − 2.2)	− 46.0
24 months (*N* = 13)	4 (30.8)	3 (23.1)	3 (23.1)	+ 8.6 (− 2.2, 16.3)	− 22.7 (− 32.7, − 12.2)	− 41.4

Presented are *n* (%) for categorical variables and median (25th, 75th percentile) for continuous variables. Participant who had bariatric surgery not included

^a^Overall change in weight from baseline. Includes all participants who had weight measured

^b^Weight change, limited to participants who lost weight

Behavior results across measurement time points are shown in Table 4. Given the small sample size, it is not possible to compare among time points, but there do seem to be positive trends in behavior measures. In particular, the overall dietary behaviors’ (i.e., REAP-S) score and the total minutes of physical activity reported per week appeared to improve over the 24 months of follow-up, with median minutes of self-reported activity per week at baseline of 100 (25th, 75th percentile 37.5, 150) compared to 180 (25th, 75th percentile 85, 242.5) minutes at 24-month follow-up

**Table 4:** Health behaviors by time point

	Baseline (*N* = 19)	3 months (*N* = 17)	6 months (*N* = 15)	12 months (*N* = 14)	18 months (*N* = 13)	24 months (*N* = 11)
REAP-S results						
REAP-S score^a^	24 (22, 27.5)	21.5 (18.8, 23.2)	21 (19, 24.5)	20.5 (18.2, 22.5)	21 (19, 22)	20 (20, 23)
Often consume fried foods	2 (10.5)	1 (6.2)	0 (0.0)	0 (0.0)	0 (0.0)	1 (9.1)
Often consume sweets	7 (36.8)	0 (0.0)	1 (6.7)	2 (14.3)	0 (0.0)	0 (0.0)
Often consume sugary drinks	13 (68.4)	1 (6.2)	2 (13.3)	0 (0.0)	0 (0.0)	1 (9.1)
Often skip breakfast	5 (26.3)	1 (6.2)	1 (6.7)	2 (14.3)	0 (0.0)	1 (9.1)
Bring food from home, days per week	4 (2, 5)	4.5 (3, 5)	5 (3, 5)	4 (3, 4.8)	4 (3, 5)	4 (3, 5)
Buy food at work, days per week	1 (0, 1)	0 (0, 0.2)	0 (0, 0.5)	0 (0, 1)	0 (0, 1)	0 (0, 1)
Minutes of self-reported activity per week^b^	100 (37.5, 150)	195 (50, 285)	190 (30, 392.5)	105 (0, 285)	100 (45, 250)	180 (85, 242.5)
Get recommended level of exercise (150 min MVPA/week)	8 (42.1)	4 (21.1)	6 (31.6)	6 (31.6)	3 (23.1)	5 (45.5)

Presented are *n* (%) for categorical variables and median (25th, 75th percentile) for continuous variables. Participant who had bariatric surgery not included

^a^REAP-S score ranges from 13 to 39. Lower scores represent healthier choices

^b^Computed by adding responses to all three survey questions (minutes per week walking, doing moderate activity, doing vigorous activity)

The most commonly selected goal was getting brisk activity (Table 5), with 14 participants selecting this goal. Participants had the opportunity to change goals or select to continue with their previous goal; thus, the number of participants for brisk activity is interpreted as follows: this goal was selected by 14 individuals at 74 coaching sessions. These 14 participants responded to prompts about their progress on this goal in 667 weeks and reported successfully meeting their brisk activity goal 49% of the time. Several other goals were selected by five or more participants. All goals except low-fat dairy were selected by at least one participant, though increasing whole grain intake and decreasing total calories were each selected by only one participant. All pilot participants had a physical activity goal (i.e., brisk activity or steps) at some point throughout the program. The percent of participants who selected each goal and who reported achieving the goal at some point during the 24 months of follow-up varied considerably across goals.

**Table 5:** iOTA goal selection and completion

Goal	*N* participants	Times chosen at coaching sessions	Weeks participants responded to goal prompt	Average goal success^a^ (%)
All goals	19	309	2965	56.9
Brisk activity	14	74	667	49.1
Steps	11	39	375	31.2
Healthy breakfast	11	35	345	68.5
Sugary drinks	9	32	347	78.0
Purchased food—meals at home	7	23	218	71.2
Screen time snacking	7	18	195	79.7
Fruits and vegetables	7	15	166	45.5
High-calorie snacks	6	19	208	65.0
Self-monitoring of diet	5	6	62	13.8
Vegetables	5	5	68	83.0
Portion control	4	14	81	87.8
Free food—limit at work	4	6	68	76.0
High-fat meats	3	9	26	57.1
Purchased food—limit at work	3	5	51	97.0
Added calories	2	4	46	24.6
Purchased food—healthy meal choices	2	3	32	90.9
Total calories	1	1	6	0.0
Whole grains	1	1	4	100.0
Low-fat dairy	0	0	0	N/A

Participant who had bariatric surgery not included

^a^As reported in SMS text message responses

Overall, participants reported very high satisfaction with the program; mean scores for each item were near 5, indicating strong agreement with the following statements: the advice given in my coaching session was helpful (mean = 4.9); the information given was relevant (mean = 4.8); the health coach was motivating in terms of getting/staying physically active and/or eating nutritiously (mean = 4.9); the health coach was understanding and supportive (mean = 4.9); the overall level of service and support was good (mean = 4.8); the text message check-ins have helped me to be active and/or eat nutritiously (mean = 4.6); and the handouts helped me to be active and/or eat nutritiously (mean = 4.4).

## Discussion

This pilot study demonstrates participant engagement with and acceptability of an SMS text-based iOTA among a group of office workers. While retention was quite high (17 of 20, 85%) over the planned 3-month pilot, engagement was further demonstrated by the 14 (70%, including the participant who had bariatric surgery) who continued to participate in coaching for 24 months (21 months longer than initially intended). Further, the pilot demonstrated high response rates to iOTA texts and very strong satisfaction with the program.

Few studies have tested interventions in low-wage working populations, and in particular, few have focused on interventions that can be disseminated and sustained. Due to the enthusiasm of participants, this pilot is considerably longer than many pilots of text-based interventions; for example, a review of text-messaging interventions to prevent cardiovascular disease found pilot studies ranged from 1 to 4 weeks [46]. Several studies looking at engagement with technology-based interventions using media besides texting and weight outcomes have demonstrated the importance of engagement with the intervention in promoting weight change. One study found a significant association between weight loss and engagement with an online weight loss program [47]. Another found those with higher engagement in a mobile phone-based coaching intervention, which included intensive health coaching via live video, phone, and text message through an app, lost more weight than those with lower levels of engagement [48]. An intervention delivered through mobile phones, to pregnant women [49], which incorporated the self-assessment and goal setting components into the mobile platform in addition to goal setting, found 45% completing the assessment step, 25% completing the goal setting step, and 23% competing the self-monitoring steps, with relationships between weight outcomes and engagement varying by BMI [50]. Therefore, though the impact on weight in the current pilot was not as clear, the finding that not only did participants want to continue in iOTA but that they were highly engaged is encouraging. Involving both SMS text messaging and in-person coaching may have led to higher engagement, which has the potential to increase intervention impact. Further, this study was conducted among a group of employees working together, which could enhance social support; iOTA was deigned to be nested within a workgroup intervention, which is why the larger trial evaluating iOTA is cluster randomized at the workgroup level.

While not designed or powered to determine efficacy, preliminary findings suggest a trend toward increase physical activity and some amount of weight loss among more than one quarter of participants. Three (23%) of the 13 participants remaining in the study at 24 months lost 5% of their body weight, which is in line with findings from a 2015 review of real-world diabetes prevention programs. This review found the percent of participants successfully achieving a 5% weight loss ranged from 20 to 64% and a mean weight loss across studies of 10.1 kg (22.3 lbs.); weight change in the current study was a gain of 4.8 pounds. This should be interpreted with caution given recent findings from a large trial of an intervention among middle- and low-wage workers in a warehouse setting, which found positive outcomes in terms of self-reported health behaviors, but no benefits in clinical outcomes [51]. However, other interventions employing text messages to promote weight loss have been successful in patients with prediabetes [52].

Important lessons related to participant expectations learned in the course of this pilot study informed the larger, subsequent evaluation of iOTA. For example, the pilot study began over the winter holidays, a time which is commonly related to weight gain [53–57]. This may have driven enthusiasm among participants and encouraged longer participation, heightening the importance of a randomized study, where participants in both intervention and control arms begin the study at similar times of year, so such seasonal effects do not impact conclusions related to intervention engagement or efficacy. Another important lesson was related to scales used for self-monitoring of weight. For self-monitoring, in particular for responding to the weekly weight check-in texts, participants were encouraged to use either home scales or a scale provided by the research team that was left at the worksite and to use the same scale each time. However, when weight was assessed using a study scale (calibrated monthly and brought on-site for study measurements), the weights were different from those participants expected based on their ongoing self-weighing, perhaps as participants were weighing at different times of day or in different clothing. This led to disappointment and discouragement, as participants thought they were losing weight based on self-monitoring, but may not have seen the same success in more-accurate measurement sessions. This suggested the importance of calibrating scales provided to worksites for self-monitoring, providing instructions on weighing at consistent times of day in similar amounts of clothing, as well as managing expectations with participants up-front about the potential for different readings on different scales and measurement issues.

This pilot had additional implications for conducting the larger study, related to goals and message communication. During the initial phase, the pilot participants received four reminder messages each Monday: one to remind them they would be asked about their weight and then one for each of their goals reminding them what their goal was and that they would be asked to report on that goal the next day (Tuesday). Since participants felt this was an excessive number of messages, the reminder system was adjusted such that participants started receiving just one message on Mondays reminding them they will be asked to report their weight and goals on the following day and that if they would like a reminder of what goals they had selected, they could request this by text. The pilot of iOTA also led to a change in goal selection instructions. Initially, participants were required to pick three goals, but based on feedback from participants indicating this could be too much to work on at once, and that they may be more successful focusing on a smaller number of goals, the instructions were modified to encourage participants to pick *up to* three goals. While a detailed description of the adaptation of an evidence-based intervention [39, 40] to develop the current iOTA has been described elsewhere [38], this pilot work was critical to preparing iOTA for evaluation in a randomized trial.

This study makes an important contribution to the literature in demonstrating the potential for this type of technology-based intervention to reach participants and for participants to be highly engaged. Further, lessons learned can inform a larger trial as well as other programs targeting behavior change among low-wage employees, particularly those utilizing SMS text messaging. These lessons include incorporating ongoing methods to check participant engagement. For the current pilot, this took the form of automatic generation of reports to alert coaches when participants indicate not meeting their goals several weeks in a row, those not responding to text message prompts, and those gaining weight, so a coach could reach out directly, providing extra reinforcement only to those who needed it. Additionally, as mentioned, the pilot information can be helpful for future studies to advise participants about the potential for measurement error when home scales are used for self-monitoring of weight. This can help set appropriate expectations and prevent the demoralizing experience participants reported when they found out their weight had not changed as they thought it had.

As a pilot, this study is limited by the small sample size and the single workgroup. An ongoing study is currently testing the final iOTA among a larger number of workgroups and hundreds of participants using a cluster randomized design [41]. This will not only allow for efficacy testing, but will also provide additional statistical power to explore questions, such as whether engagement is associated with weight loss success, and will allow for exploration of what goals might be most strongly associated with engagement and weight loss.

## Conclusions

This pilot provides important preliminary results on high acceptability and good participant engagement with an SMS-based intervention intended for low-wage workers, with significant potential for dissemination and sustainability. Lessons learned from this pilot such as modifications to instructions for goal selection and message timing were important in enhancing the larger trial evaluating iOTA.

## Data Availability

The datasets used and/or analyzed during the current study are available from the corresponding author on reasonable request and pending appropriate human subjects and confidentiality protections.

## Abbreviations

SMS: Short message service
BMI: Body mass index
iOTA: Interactive obesity treatment approach
REAP-S: Rapid Eating Assessment for Participants
IPAQ: International Physical Activity Questionnaire
MVPA: Moderate to vigorous physical activity
